# An Integrated Dynamic Closed Loop Simulation Platform for Elbow Flexion Augmentation Using an Upper Limb Exosuit Model

**DOI:** 10.3389/frobt.2022.768841

**Published:** 2022-03-17

**Authors:** Ratna Sambhav, Shreeshan Jena, Ankit Chatterjee, Shubhendu Bhasin, Sushma Santapuri, Lalan Kumar, Suriya Prakash Muthukrishnan, Sitikantha Roy

**Affiliations:** ^1^ Department of Applied Mechanics, Indian Institute of Technology Delhi, New Delhi, India; ^2^ Department of Electrical Engineering, Indian Institute of Technology Delhi, New Delhi, India; ^3^ Department of Physiology, All India Institute of Medical Sciences, New Delhi, India

**Keywords:** exosuit, human in-loop biomechanics, human-machine interaction, musculoskeletal simulation, assistive device, symbiotic biomechanics

## Abstract

Wearable robotic devices are designed to assist, enhance or restore human muscle performance. Understanding how a wearable robotic device changes human biomechanics through complex interaction is important to guide its proper design, parametric optimization and functional success. The present work develops a human-machine-interaction simulation platform for closed loop dynamic analysis with feedback control and to study the effect of soft-robotic wearables on human physiology. The proposed simulation platform incorporates Computed Muscle Control (CMC) algorithm and is implemented using the MATLAB -OpenSim interface. The framework is generic and will allow incorporation of any advanced control strategy for the wearable devices. As a demonstration, a Gravity Compensation (GC) controller has been implemented on the wearable device and the resulting decrease in the joint moments, muscle activations and metabolic costs during a simple repetitive load lifting task with two different speeds is investigated.

## 1 Introduction

Exoskeletons and exosuits are the terms used interchangeably to refer to a class of wearable assistive devices which work in tandem with the human body to provide assistance. The assistance provided by these human joint force amplifiers can augment, reinforce or even restore human performance. Muscle strength augmentation for workers or soldiers (Wehner et al., 2013), assistance to elderly generation in activities of daily living (ADLs), i.e., the collective tasks carried out by an individual such as picking up an object, walking, climbing, personal hygiene tasks, (Wu et al., 2019), muscle performance restoration in paraplegic patients (Dinh et al., 2017), are few of the many applications of these devices. A review of existing devices and research approaches suggests that the exosuit design depends on some crucial developmental aspects: the user intention estimation, assistance moment estimation, and the comfort of force transmission (Chiaradia et al., 2018; Lotti et al., 2020a; Lotti et al., 2020b; Zhang et al., 2021). As the experimental evaluation of these aspects on a physical setup can be tedious and accompanied by safety concerns, there is a requirement for the development of an integrated simulation framework where a virtual human model coupled with an external augmentation device can be tested and subsequently optimized. Prior simulation also enables device optimization based on the parameters that are difficult to measure experimentally, such as the interaction forces, joint reaction forces, etc. The simulation framework requires an integrated environment where, along with musculoskeletal dynamics, intention estimation using a brain control interface (BCI), controller implementation and actuator modules can be collated to study the resulting physiological parameters on the virtual human model. Finite element analysis (FEA) has been used in recent studies for estimating the user comfort, and can also be conceptualized as an additional module within the framework (Zhang et al., 2021). The initial challenges with the development and implementation of musculoskeletal models have been outlined in recent studies (Thelen et al., 2003; Mansouri and Reinbolt, 2012; Akhavanfar et al., 2019).

In recent literature, Zhang et al. employed an inverse dynamics and optimization-based technique using the Anybody Inc. software (Rasmussen, 2019) to observe the effects of different assistive strategies on the reduction in joint moment and muscle impulse (Zhang et al., 2021). They used a “muscle recruitment algorithm” to calculate the muscle activations and the interaction forces at all the contact points between the exoskeleton and human body (Zhang et al., 2021). A feedback method is adopted by Stollenmaier et al. where open loop and closed motor commands together drive a forward dynamics model (Stollenmaier et al., 2020). The command generator drives the open loop motor command using the input trajectory, inverse dynamics and optimization steps (Stollenmaier et al., 2020). Subsequently, the closed loop motor command is obtained after multiplying the error in desired and current values of muscle fiber lengths and contraction velocities with the proportional and derivative feedback terms. Models in the recent literature relate motor commands to changes in object (limb) states (i.e., position and velocity) to influence the signals in a physiologically-accurate and predictable manner (Thelen, 2003; Bhanpuri et al., 2014; Lee and Umberger, 2016).

In this paper, the position and velocity data of the joint from the forward dynamics are fed back, so that the inverse dynamics-based motor command generator can compensate for the error terms. The present simulation framework implements the open-source platform OpenSim (Delp et al., 2007; Seth et al., 2018), that is widely established for musculoskeletal simulations. MATLAB is the base platform for this simulation framework, and incorporates the multibody dynamics of OpenSim (using the Application Programming Interface (API) library) along with the BCI, optimization, control, actuator and physiological modules. The hypothesis considered for the present study is that the use of an external assistive device can help in reducing the joint moment, muscle activations and metabolic cost associated with the activity (elbow flexion in the present study).

## 2 Methods

### 2.1 System Architecture

**FIGURE 1:**
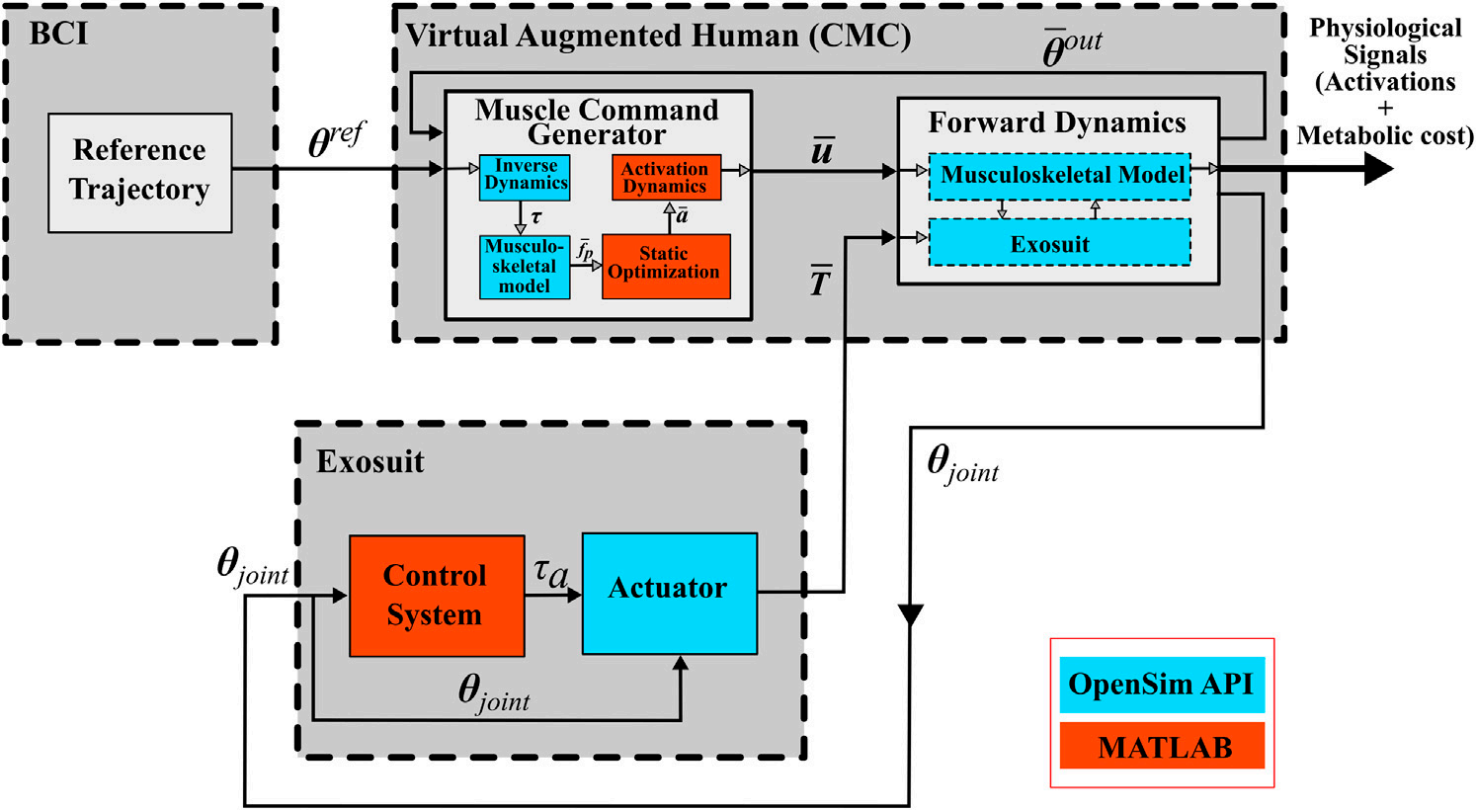
The overall architecture of the proposed simulation framework, where virtual human represents the user wearing the exosuit. $\theta^{ref}$ represents the reference trajectory generated by the user’s mind estimated from BCI. ${\bar{\theta }}^{out}:\;$ output joint kinematics, τ : joint moment, ${\bar{f}}_{p}:$ the input parameters for static optimization, $\;\bar{a}:$ muscle activations $\bar{u}$ : motor commands (muscle excitations), $\bar{T}$ : tensions in the extensor and flexor cables, $\tau_{a}$ : desired actuator moment at elbow, and $\;\theta_{joint}$ : output position of elbow joint. The physiological signals (muscle activations and metabolic costs) are the output of this simulation framework.

The present simulation framework creates a digital model of the human musculoskeletal system integrated with an external assistive device. The overall system architecture is divided into the brain computer interface (BCI), the musculoskeletal biomechanics, actuator and controller modules. This overall Computed Muscle Control (CMC) architecture (the “Virtual Augmented Human”) is presented in Figure 1, showing the flow of information between each module of the framework. Based on the reference trajectory $\left(\theta^{ref}\right)$ , the muscle command generator in OpenSim computes the human muscle excitation signal ( $\bar{\mu }$ ) using a series of steps – *1*) inverse dynamics, *2*) musculoskeletal model, *3*) static optimization, and *4*) activation dynamics. The muscle excitations are combined with the exosuit’s actuator signal that is generated by the controller implemented in MATLAB. The combined signal (muscle excitation + external actuator) is then sent as an input to the forward dynamics block in OpenSim which generates the human motion and computes the metabolic cost. The joint angles from the motion are fed back to the controller to close the loop. This closed-loop system involves continuous exchange of information between MATLAB and OpenSim and is possible due to the OpenSim application programming interface (API) commands. The functional blocks within these modules are the following:

#### 2.1.1 Muscle Command Generator (MCG)

The purpose of MCG is to use the error between the desired and actual trajectories to calculate the required excitations in the muscles such that the trajectory error is also minimized. The MCG also uses the position and velocity feedback from the simulated output trajectory. Inverse dynamics simulator calculates the joint moments from the input kinematics, followed by the static optimization to solve for the muscle redundancy problem and obtain the muscle activations. Finally, the equations involved in activation dynamics are used to calculate the corresponding muscle excitation values. The inverse dynamics and musculoskeletal models within the MCG use the OpenSim API commands while the static optimization and activation dynamics are carried out entirely using MATLAB algorithm. The MCG functionality is explained using the following:

***Inverse Dynamics*:** The desired joint trajectory as well as the error between the simulated and desired trajectories is used to calculate the joint moments (in the presence or absence of an external force) such that the joint moment,

$$\tau =M\ddot{\theta }-G\left(\theta \right)-C\left(\theta ,\dot{\theta }\right)-A\left(\theta ,\dot{\theta },t\right)$$ (1)

Where, $A\left(\theta ,\dot{\theta },t\right)$ term represents any externally applied force or moment corresponding to the position ( θ ), velocity ( $\dot{\theta }$ ) and time ( t ). The terms G(θ) for gravity and $C\left(\theta ,\dot{\theta }\right)$ for centripetal and Coriolis, are calculated from the current values of θ and $\dot{\theta }$ . However, in the inertial term $M\ddot{\theta }$ , the acceleration at the joint is the sum of desired acceleration ( ${\ddot{\theta }}_{des}$ ) and the feedback error terms such that,

$$\ddot{\theta }={\ddot{\theta }}_{des}+k_{p}\left(\text{Δ}\theta \right)+k_{v}\;\left(\text{Δ}\dot{\theta }\right)$$ (2)

Where $k_{p}$ and $k_{v}$ are the feedback gains for the position and velocity errors respectively. The k_p_ and k_v_ values from Thelen et al. (2003) were 400 and 40 respectively. However, for the present framework the set of values of 900 and 60 (for k_p_ and k_v_ respectively) provided the best error compensation, especially for the simulations where the exosuit provided assistance. Eq. 2 allows for the compensation of the errors in position and velocity of the joints. In case of assistance, the term $A\left(\theta ,\dot{\theta },t\right)$ becomes non-zero, resulting in reduced joint moment.

***Musculoskeletal Model:*** The musculoskeletal model comprises a forward simulation of the arm26 musculoskeletal model, which is well documented in the open literature (Mansouri and Reinbolt, 2012; Gastaldi et al., 2020). The Hill-type muscle model has been implemented to execute the resulting joint motion from the input controls. The Hill-type muscle model has been used for the forward simulation within the present framework to present the net force in the muscles as

$$F=\;F_{m}\left[f\left(l\right)f\left(v\right)a\left(t\right)+\;f_{p}\left(l\right)\right]\text{cos}\left(\emptyset \left(t\right)\right)$$ (3)

Where, $F_{m}$ is the maximum isometric force of the muscle, f(l) , f(v) and $f_{p}$ are the generic force-length, force-velocity and passive elastic force-length curves. The activation input is represented as a(t) and the muscle pennation angle by ∅(t) . The maximum isometric forces, muscle moment arms, muscle fiber lengths, optimal fiber lengths, fiber contraction velocity and maximum fiber contraction velocities are obtained from OpenSim using the API commands, and further used to calculate the f(l) and f(v) parameters in MATLAB using the force-length and force velocity relationship from the Thelen. 2003 muscle model (Thelen, 2003).

***Static Optimization:*** The individual muscle forces are calculated by optimizing the distribution of the activation signals ( $a_{i}$ ) among the muscles. The above Hill-type musculoskeletal model in Eq. 3 is used to calculate the muscle forces. The optimization function uses the objective function J of the form

$$J=\;\overset{6}{\underset{i=1}{\sum }}a_{i}^{2}\;$$ (4)

Where, *i* represents the muscles (biceps long, biceps short, brachialis, triceps long, triceps medial and triceps lateral), involved in the simulation. The objective function ***J*** is chosen as a square of the activations, as previous literature suggests that the quadratic criterion provides a reasonable to good estimate of the muscle activations (when compared to the EMG signals) (Happee, 1992). The optimization function implements the constraints by equating the moment calculated from the inverse dynamics to the moment calculated as a product of the individual muscle forces and respective moment arms about the joint. Optimization is implemented using *fmincon* function (*large scale interior point* algorithm) in MATLAB, where Eq. 4 was set as the minimization function, subject to the equality constraints obtained from Eq. 1 and the following:

$$\tau =\overset{6}{\underset{i=1}{\sum }}d_{i}F_{i}$$ (5)

And the inequality constraints

$$0\leq a_{i}\leq 1$$ (6)

Where τ is the elbow joint moment, $d_{i}$ is moment arm of the muscles about elbow joint, $F_{i}$ is the force exerted by individual muscles and *a*_*i*_ is the activation corresponding to the *i* th muscle.

***Activation Dynamics:*** The forward dynamics simulation in the system framework requires the muscle control signals (excitations) as input. The individual muscle excitations are calculated from their activation values using the activation dynamics equation (Thelen, 2003) shown below:

$$\frac{da}{dt}=\;\frac{u-a}{\kappa_{a}\left(a,u\right)}$$ (7)

Where, $\kappa_{a}$ *(a,u)* is the time constant whose magnitude depends upon whether the muscle activation is increasing or decreasing.

$$\kappa_{a}\left(a,u\right)=\;\{\begin{array}{c} \kappa_{act}\left(0.5+1.5a\right)\;\;\;\;\;,u>a\\ \kappa_{deact}/\left(0.5+1.5a\right)\;\;\;\;\;,u\leq a \end{array}$$ (8)

The parameters *a* and *u* are the muscle activations and excitations, respectively. The parameters $\kappa_{act}$ and $\kappa_{deact}$ denote the activation and deactivation time constants respectively.

#### 2.1.2 Forward Dynamics

The forward dynamics (a sub-component of the CMC algorithm) uses OpenSim API commands to determine the joint reaction forces and exosuit-human interaction forces, along with the error between the desired and output joint trajectories, thus refining the accuracy of estimated muscle excitations.

#### 2.1.3 Wearable Exosuit

##### Control System

A gravity compensation (GC) based control strategy is implemented in the assistive device. In the present study, the assistive moment at the joint is directly proportional to the angle between the forearm and the direction of gravitational force at the elbow joint. In the **assisted** condition (actuator ON) the exosuit provides assistance to the elbow joint, while in the **unassisted** condition (actuator OFF) the exosuit doesn’t provide any assistance to the elbow joint. The GC controller compensates for the gravity dependent component of the moment acting at the elbow joint, given by:

$$\tau =mgl_{c}sin\theta +\;Mgl_{l}sin\theta \;$$ (9)

Where θ is the angle between forearm and the vertical line, m  is forearm mass, M is mass of extra load, $l_{c}$ is distance of center of mass of forearm from the elbow joint, and $l_{l}$ is the distance of extra load from the elbow joint. The GC strategy is simliar to that adopted by Dinh et al. (Dinh et al., 2017). In a physical exosuit, θ can be obtained from the inertial measurement units (IMUs) attached to the forearm. However, in the simulation environment this information is taken from the measured output elbow joint angle.

##### Actuator Module

This module is used to calculate the forces in the extensor and flexor cables such that the desired moment can be transmitted to the elbow joint. In the simulation, these forces are directly generated using the path actuators (OpenSim API). The actuator module is based on the current value of moment arms of flexor-extensor cables, in addition to the magnitude and direction of the desired moment (to be transmitted to the elbow joint), and computes the value of the tension required in the cables. These forces in the simulation are directly applied to the path actuators in the forward dynamics block.

For Actuator moment >0:

$$T_{1}=\tau_{a}/r_{1},$$ (10)

$$T_{2}=0,$$ (11)

For Actuator moment <0:

$$T_{1}=0,$$ (12)

$$T_{2}=\tau_{a}/r_{2},$$ (13)

Where $T_{1}$ is the tension in flexor cable, $T_{2}$ is the tension in extensor cable, $r_{1}$ is the moment arm of flexor cable, and $r_{2}$ is the moment arm of extensor cable.

#### 2.1.4 Brain Computer Interface (BCI)

The functionality of BCI module in the simulation environment is to test and validate the different methods of desired motion estimation in sync with the other modules of exosuit like controller and actuator by taking data from a pre-recorded EEG dataset. In the current simulation study though, the control system uses gravity compensation control which only requires the joint angle data as input, and the BCI module comprises a ready-made reference trajectory as input to the simulation.

### 2.2 Simulation Framework

We developed a MATLAB based platform in the present study, to access the OpenSim functionalities using the OpenSim API, and perform the forward and inverse dynamics of the musculoskeletal system. MATLAB provided the requisite functionalities for the calculations involving controller, actuator and muscle activations. The simulation platform is versatile and provides integration with multiple software, and subsequently other utilities such as brain machine interface and finite element analysis for study of force interaction can be interfaced into the simulation. The simulation framework involved the OpenSim Inverse Dynamics Solver as well as the Forward Integrator (using a Runge-Kutta-Merson integrator) by default. The static optimization carried out in MATLAB uses a *large scale interior point* algorithm. The algorithms for controller and actuator modules used MATLAB for calculations. A sampling rate of 121 Hz was used for the joint angle data from 0 to 1 s. The simulation presents robust results with variations of step-sizes between 0.004s < Δ t < 0.02 s. The robustness of the present simulation framework can be further deduced from the low error between the reference and forward dynamics trajectories when the motion (elbow flexion from 0° ° to 90° ° ) was simulated at different rates (2×, 1×, and 0.5× speeds), as well as when a rapid change or intermittent perturbation were introduced.

### 2.3 Physical Model and its Digital Counterpart

**FIGURE 2:**
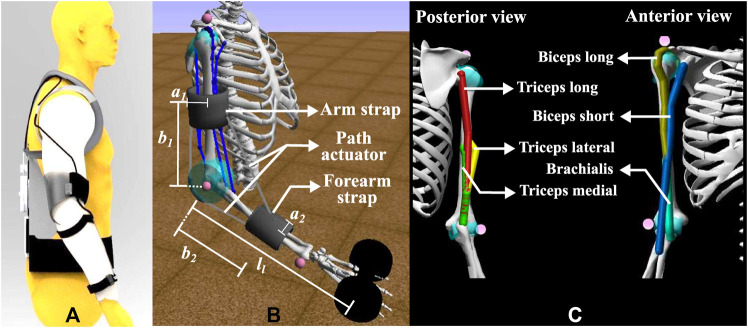
The **(A)** CAD model illustration of the exosuit placement on the human, **(B)** corresponding setup (simplified) for the elbow flexion simulation using the MATLAB-OpenSim framework, and **(C)** the muscles included in the present model.

Figure 2A illustrates through CAD model, the physical design of the exosuit on a dummy human model. Exosuit design consists of the following units: *1*) wearable fabric and straps, *2*) actuator unit, *3*) controller unit, *4*) cable routing, and *5*) battery pack. As shown in Figure 2A, the actuator and controller units with battery are placed on the back of the human. Bowden cables (agonistic and antagonistic) are routed along the lines of minimum extension, covered with sheath, to the arm straps. These Bowden cables are attached between the upper arm and forearm straps to form the flexor and extensor pairs. Based on the moment and rotational direction of motor, tension is generated in the flexor or extensor cable.

**FIGURE 3:**
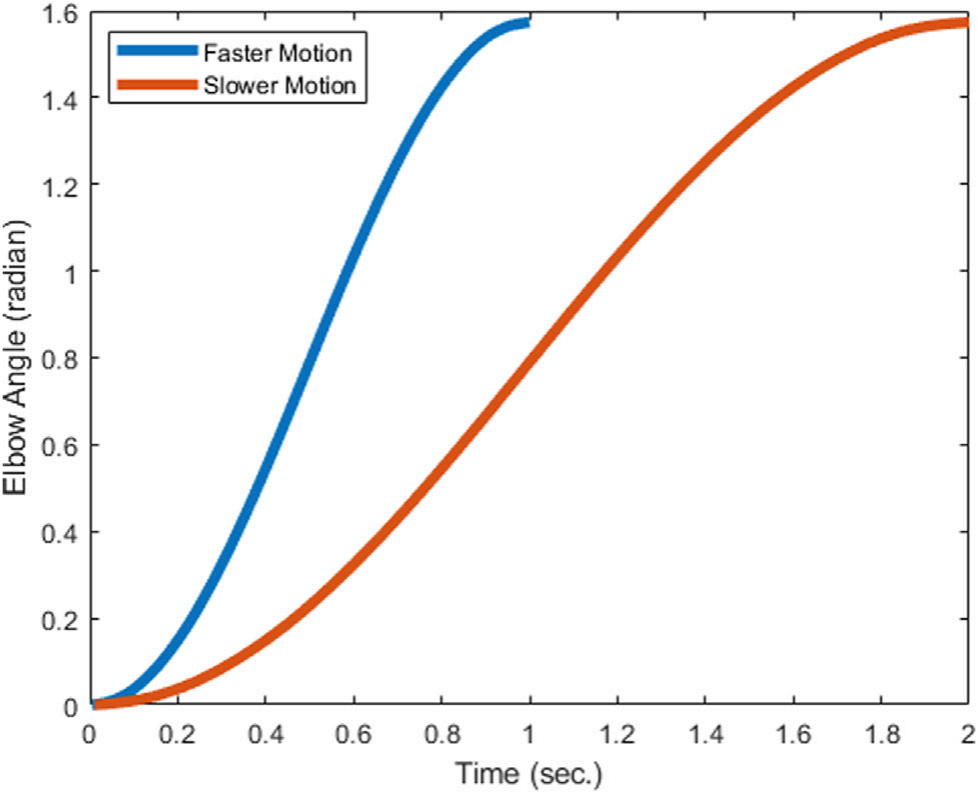
Reference trajectories are minimum jerk trajectories from 0 to 1.57 rad in 1 and 2 s respectively for faster and slower motion.

For its digital counterpart, we used a right upper body model “arm26” as the musculoskeletal model, representing the digital human, as shown in Figure 2B. This simplistic model provides two degrees of freedom, one at the elbow and another at the shoulder, and the plane of motion is restricted to the sagittal plane. This model has six muscles of the elbow, i.e. biceps long, biceps short, brachialis, triceps long, triceps medial and triceps lateral. The muscles for shoulder joint are omitted and the shoulder degree of freedom has been constrained, as the present focus is the study of the activation, joint moment and metabolic cost parameters of the elbow joint with and without assistance. Further, the negligible contributions of the muscles around the shoulder joint have been verified by using a separate upper-limb musculoskeletal model, the Dynamic Arm Simulator (DAS), which has 11 degrees of freedom and 138 muscles about the shoulder and elbow joints. The DAS model was also constrained similar to the arm26 model, permitting only the elbow flexion DOF. An input trajectory for the elbow flexion similar to the one used in this paper was fed to the DAS model and the static optimization tool in OpenSim was used to calculate the muscle activations. The input reference trajectories (slow and fast) for the simulation are presented in Figure 3.

As a part of the exosuit, the CAD designs of exosuit straps were imported and attached to their appropriate locations over the digital human model. The force transmission system from the motor to the anchorage points are modeled directly as force generating elements between the two straps in the digital model. The force generating elements are the *path actuators*, which apply equal and opposite force on the segments to which they are attached. These path actuators are attached agonistically and antagonistically, in between the upper-arm and forearm straps, as shown in Figure 2B. Using the *prescribed controller*, control signals were sent to the path actuators to create the desired level of tension. All the geometrical input parameters of the model are given in Table 1.

**TABLE 1:** The geometrical parameters in the present model.

Parameter	Symbol	Value
Forearm Mass	*m*	1.53 kg
Forearm COM from Elbow Joint	*l*_*c*_	0.18 m
Moment of inertia of forearm segment about elbow joint	*I*_*xx*_ *, I*_*yy*_ *, I*_*zz*_	(0.02, 0.001, 0.02) kgm^2^
Distance of external load from Elbow Joint	*l*_*l*_	0.35 m
Arm anchorage point location w.r.t Elbow Joint	*a*_*1,*_ *b*_*1*_	(0.04 m, 0.14 m)
Forearm anchorage point location w.r.t Elbow Joint	*a*_*2,*_ *b*_*2*_	(0.02 m, 0.15 m)

#### 2.3.1 Simulated Task

The simulation was repeated for two conditions of the elbow flexion: *1*) a fast and *2*) slow flexion motion, with and without assistance from the exosuit. In the current simulation study, we constrained our analysis to elbow joint only, and locked the shoulder degree of motion (at a 0° orientation). Following are the parameters varied in different simulation iterations:

*External load in hand*: An external mass has been attached at a distance of 29 cm from the elbow joint, as shown in Figure 2. The mass of this load is varied (0, 2, 5 kg) to observe the rise in muscle activation patterns and subsequently, the reduction in the muscle activity after incorporating actuator assistance.

*Reference trajectory*: The elbow joint of the simulation model undergoes motion from an initial angular position of 0° to a final position of 90°, following a minimum jerk trajectory path for a specified time duration. As shown in Figure 3, two types of motion are used for simulation, one which achieves this trajectory in 1 s, and the other which achieves it in 2 s. The two input reference trajectories for achieving the desired orientation are shown in Figure 3.

*Metrics of evaluation*: We calculated the joint moment, activation and metabolic cost parameters from the simulation platform and observed the changes in magnitudes for different parametric variations within the simulation, i.e., varying speeds, external loads and actuator assistance (ON/OFF). The metabolic cost function for the activity has been calculated as the sum of rate of heat liberated from the body and the rate at which work is done (Umberger et al., 2003).

$$\dot{E}=\;\dot{B}+\left(\dot{A}+\dot{M}+\dot{S}+\dot{W}\right)$$ (14)

Where, $\dot{B}$ is the basal heat rate, $\dot{A}$ is the activation heat rate, $\dot{M}$ is the maintenance heat rate, $\dot{S}$ is the shortening heat rate and $\dot{W}$ is the mechanical work rate (W).

## 3. Results

### 3.1 Muscle Activations

**FIGURE 4:**
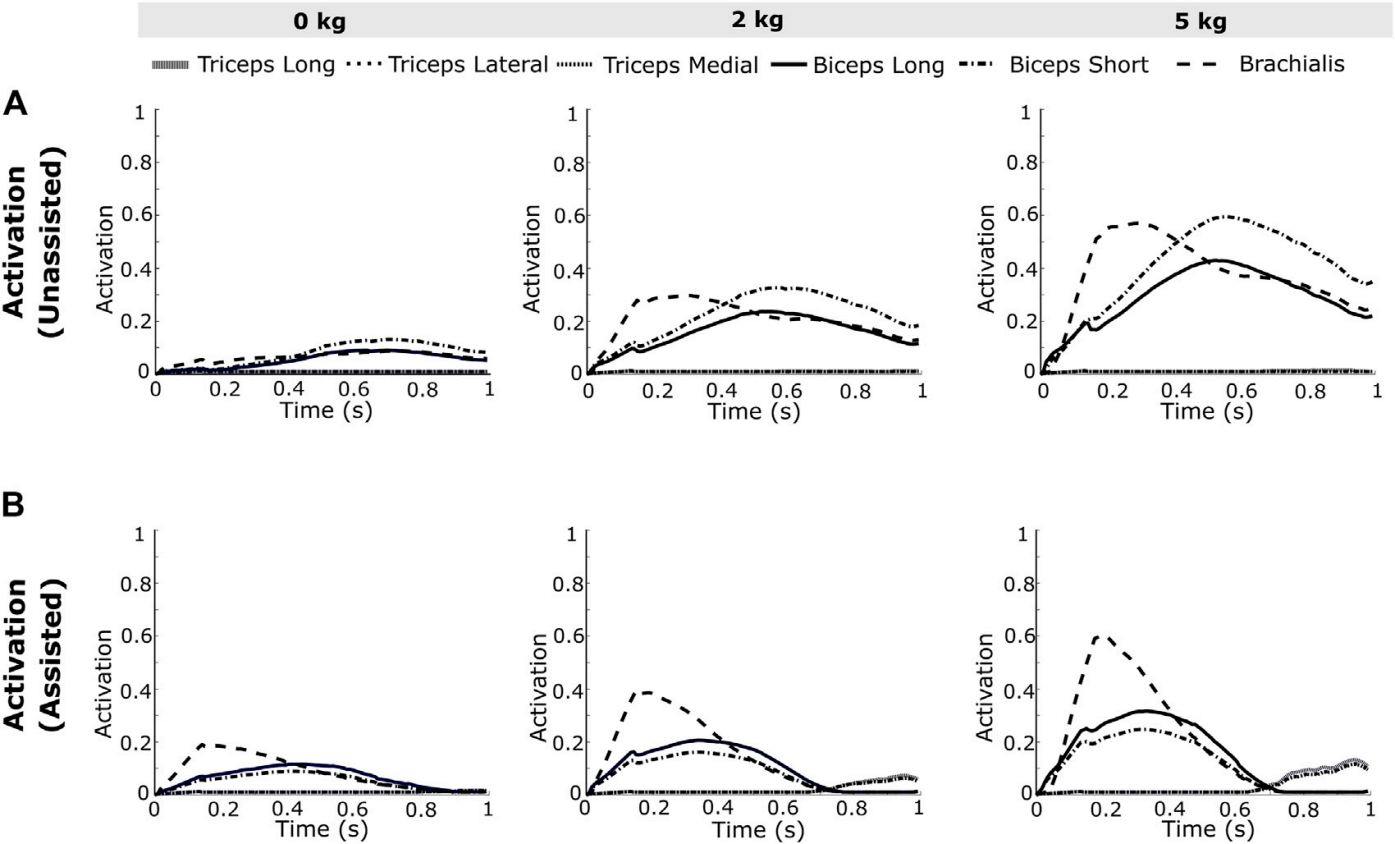
The resulting activations, **(A)** unassisted and **(B)** assisted, obtained from the simulation of the framework for the elbow flexion with no load, 2 kg load and 5 kg load at the palm illustrate a visible reduction in the muscle activations after assistance from an external actuator implementing the gravity compensation control scheme.

**FIGURE 5:**
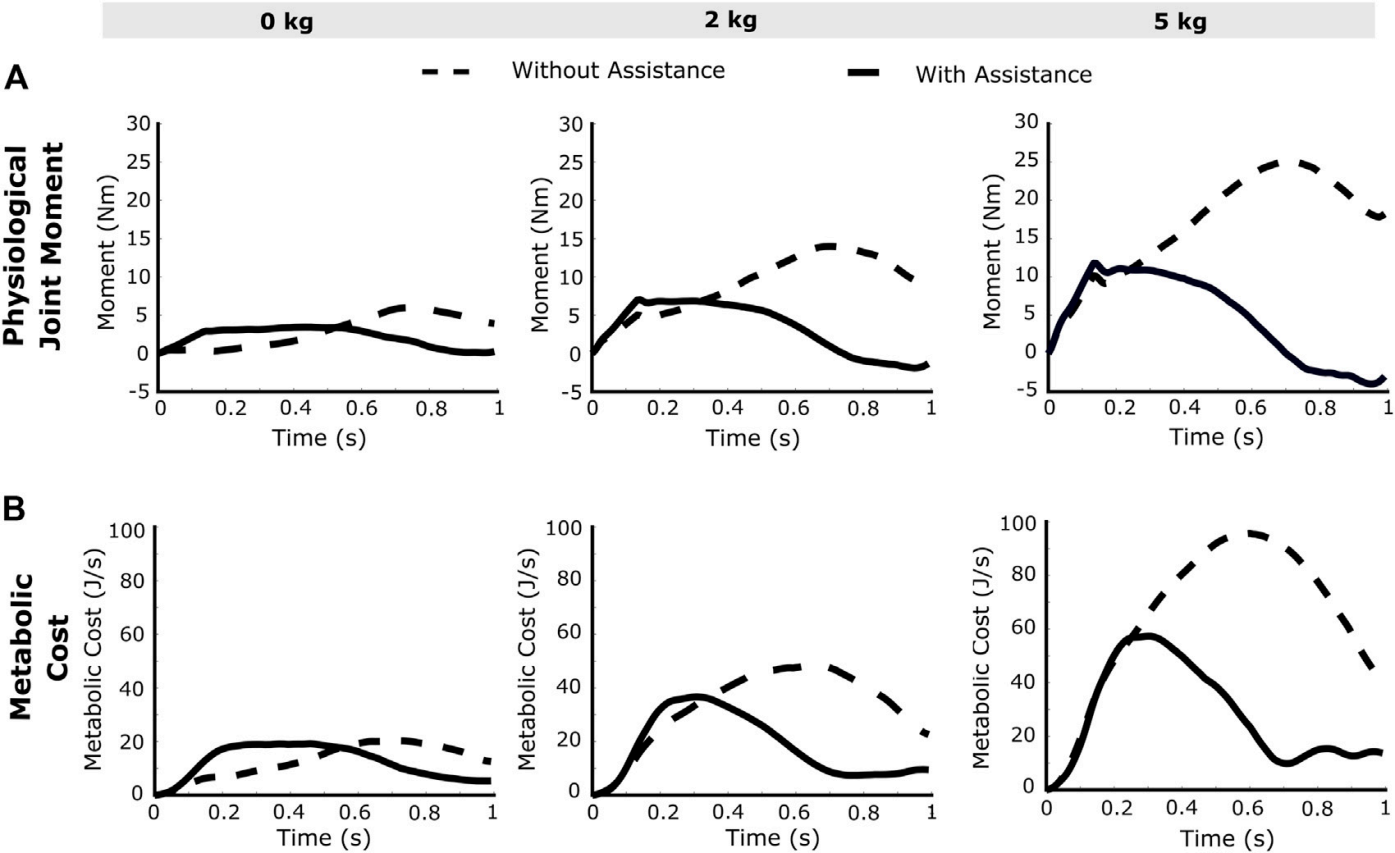
The resultant **(A)** joint moments about the elbow and **(B)** the metabolic costs calculated from the elbow flexion simulation with no load, 2 kg load and 5 kg load at the palm. The joint moment plots (with assistance) show negative magnitude owing to the activation in the antagonistic muscles.

**FIGURE 6:**
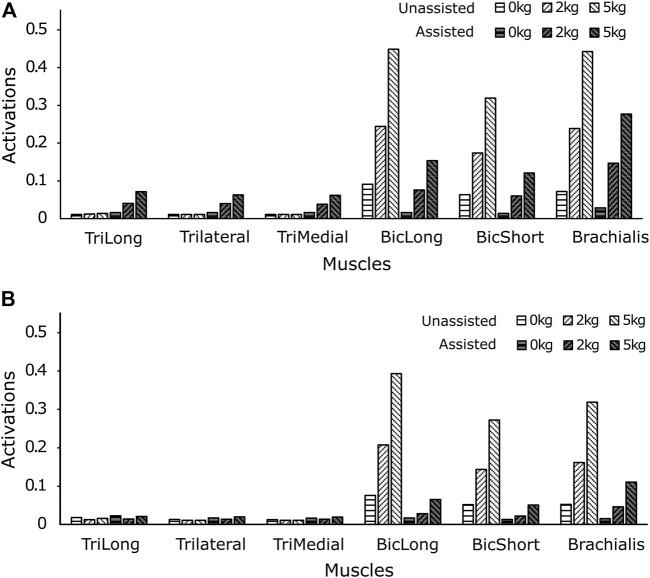
The RMS values of muscle activations for **(A)** high speed, and **(B)** low speed motion with and without assistance show a trend of reduced muscle activations with external assistance. However, at higher speed of motion the triceps group presents an increase in activations, after receiving actuator assistance.

The computed muscle activations (Eq. 4) are presented in Figures 4A,B for the high-speed trajectory with and without assistance. The drop in muscle activation levels are more prominent towards the second half of the motion (around 0.6–1 s), while some muscle groups seem to display higher activations even with exosuit assistance (Figure 4B). This unexpected increase in activations can also be observed in the joint moment plots (Figure 5A), thereby necessitating the calculation of RMS values to evaluate the overall reductions. In the case of the high-speed trajectory, the root mean squared (RMS) value of muscle activities averaged over the three biceps muscles, decreases by 73.72, 67.59 and 55.12% respectively for 0, 2 and 5 kg external loads (Figure 6). Comparatively, for the slow speed trajectory, the decrease observed are 74.2, 80.9, and 76.7%. The muscle activations of the triceps group in case of unassisted motion present a magnitude of about 0.01, irrespective of the load. However, for the condition with assistance the triceps group shows increased activations.

### 3.2 Joint Moments

The obtained joint moment increases with higher speed and increase in external mass as shown in Figure 5A. Interestingly, the joint moments show an increase in magnitude in the initial part of the motion (observed around 0.2 s), as well a negative magnitude towards the end of the motion. In case of the high-speed trajectory with assistance, the RMS value of joint moment decreases by 89.13, 74.05 and 70.78% for 0, 2 and 5 kg external loads respectively. For the slow-speed trajectory with assistance, joint moment decreases by 80.20, 91.73 and 87.56% respectively for 0, 2 and 5 kg mass in hand.

### 3.3 Joint Reaction Force

**FIGURE 7:**
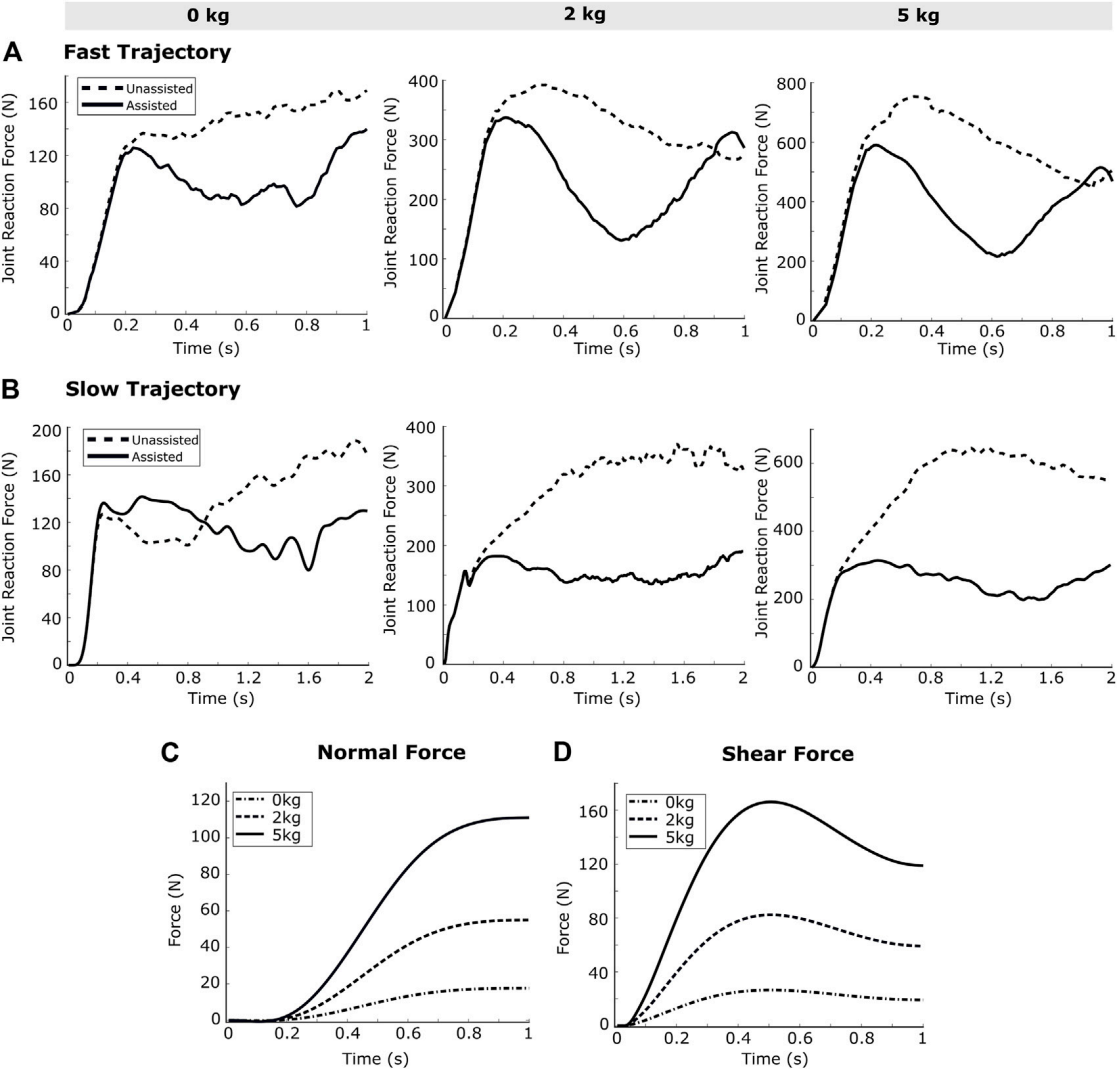
The joint reaction forces calculated from the simulation framework for the **(A)** fast trajectory and **(B)** slow trajectory show a trend of reduction while receiving external assistance. The reduction seems more pronounced in case of the slow speed trajectory. The **(C)** normal force, and **(D)** shear force at the forearm strap illustrate a proportionality between the external load and resultant forces.

The OpenSim API commands are used to obtain the joint reaction force at the elbow. The resulting joint reaction force at elbow joint is observed to decrease in assisted case compared to the unassisted, as presented in Figures 7A,B. Further, the joint reaction forces show higher reduction for the slow-speed trajectory as compared to the high-speed trajectory. For the high-speed trajectory, 28.4, 25.6 and 30.83% of decrease has been recorded respectively for 0, 2 and 5 kg external loads. For the slow-speed trajectory, 17.2, 49.1 and 51.7% of decrease have been observed.

### 3.4 Interaction Force

The normal and shear forces acting on the exosuit strap are calculated by using the OpenSim API commands. Subsequently, the results show that the normal and shear forces applied by the exosuit strap on the forearm increase with the external mass and the trend remains unchanged for different speeds of elbow joint motion (Figures 7C,D). The interaction forces calculated in the present work are dependent on the cable tensions, which in turn depend upon the actuator torque components computed by the controller. The present framework implements a gravity compensation controller which calculates the actuator torque component on the basis of the elbow angle ( θ ) only (the $\dot{\theta }$ and $\ddot{\theta }$ components are not considered for the actuator torque calculation). As the interaction force results are dependent on θ , they present the same magnitudes for a particular joint angle, irrespective of the elbow joint rotational velocity (low speed or high speed trajectory). The peak values of normal forces are 12 N, 40 and 80 N respectively for 0, 2 and 5 kg external mass, whereas the peak values of shear forces are 25, 80 and 160°N, for both high speed and low speed trajectories.

### 3.5 Metabolic Cost

The OpenSim API functionality is used for calculating the metabolic cost (Figure 5B) of the musculoskeletal model for the targeted motion (Umberger et al., 2003). The magnitude of resultant metabolic costs of the model depend on work done by muscles and its activation and excitation values. After applying assistance, reduction in RMS value of the metabolic cost is, for high-speed trajectory, 60, 61.83 and 64.83% respectively for 0, 2 and 5 kg loads and for low-speed trajectory, it is 44.76, 71.93 and 75.89%.

## 4 Discussion

This paper presents the development of a generalized simulation framework and its subsequent application to an upper limb assistive exosuit. We implemented a gravity compensation based assistive strategy for the exosuit control, and studied the physiological benefits while executing elbow flexion. The goal of the controller is to provide external assistance such that the biological muscles have to compensate for the inertia component only (Eq. 1), thereby reducing a major portion (gravity component) of the moment required at the elbow joint and ultimately leading to a much lower joint moment. More sophisticated control strategy can be tried and tested on this general framework. The resultant distribution of activations from the DAS model also showed that the muscles surrounding the shoulder joint present negligible activations. Thus, the muscles that have been omitted about the shoulder joint have negligible influence on the rest of the muscle forces and the muscle force distributions from the arm26 model are considered to be independent of shoulder muscle forces (for the present configuration of simulation).

The maximum elbow acceleration for low and high speed motions are 2.5 and 8 rad/s^2^ respectively, thereby implicating a larger proportion of the gravity component in the former (Eq. 1). As a consequence, this results in a higher reduction of the joint moment for the low-speed trajectory. For higher external load values in the high-speed trajectory (Figure 5A), the joint moment attains a negative magnitude towards the end of trajectory. This is due to the negative moment required at the joint to compensate for the high deceleration (−8 rad/s^2^) and maintain the desired trajectory. In this situation, the antagonistic muscles (triceps group) present relatively higher activations (Figure 4B) to counteract the excess of the assistive moment at the elbow joint. The muscle recruitment and the change in their activation levels can be understood by inferring the joint moment variation (Figures 4, 5A). Figures 6A,B shows the RMS values of muscle activations for different conditions of loading, speed and assistance. However, even after considering the increase in triceps muscles’ activation values, the overall human (muscle) effort decreases with external assistance.

The joint reaction force is another important parameter to quantitatively evaluate the beneficial effects of an assistive device, as an increased joint reaction force can be harmful to the joint. While exoskeleton devices have a “hard” joint corresponding to the biological joint(s) to distribute joint reaction forces, the soft exosuit envisioned in the present work does not have any such joint. Interestingly, the simulation results illustrate that there is a reduction in the joint reaction force when external assistance is provided (Figure 7). As shown in Figure 7, there is a higher reduction in the resultant joint reaction force at elbow (after exosuit assistance), with the increase in external load and the speed of motion.

The interaction force between the exosuit strap and the human limb is an important parameter in the design of the exosuit. The dimensions of straps and the padding material to be used in the strap, can be calculated based on the force applied by the strap to the body, in order to bring the contact pressure within the required tolerance (Huysamen et al., 2018; Yandell et al., 2020; Kozinc et al., 2021). The simulation results have important implications for physical design of the suit, and while 80 N of normal force is well below the maximum tolerable force, 160 N of shear force may cause the forearm strap to slide over the forearm.

The metabolic cost plots presented in Figure 5B depict a high reduction in metabolic cost, and this can be attributed to the consideration of only six muscles about a single joint. Considering a healthy human anatomy, the actual experimental metabolic cost reductions may be of a different magnitude as compared to the simulation results. The present musculoskeletal model used in the simulation framework presents some limitations and may not corroborate clinical data due to the following factors: *1*) incorporation of only six muscles about the elbow joint and omitting the muscles at the shoulder and the forearm, *2*) the metabolic cost reduction values are those calculated considering these six muscles only. We have used the arm26 musculoskeletal model in the present study, whereas a more realistic simulation should consider a full-body model with all muscle definitions. However, the MATLAB-OpenSim framework provides easy access and utilization of multiple musculoskeletal models. Further, the force interaction between the actuator straps and the human twin may be carried out with the addition of a FEA module. The simulation framework described in this paper utilizes the best features of OpenSim and MATLAB to develop a system that acts as a digital model of the human physiology. Overall, the reduction in effort of the human muscle may be estimated using the framework, thereby indicating the efficiency of an exosuit implementing the proposed control strategy.

## Data Availability

The original contributions presented in the study are included in the article/Supplementary Material, further inquiries can be directed to the corresponding author.
